# Diagnostic accuracy of Fatty Liver Index (FLI) for detecting Metabolic Associated Fatty Liver Disease (MAFLD) in adults attending a tertiary care hospital, a cross-sectional study

**DOI:** 10.1186/s40842-024-00197-2

**Published:** 2024-12-13

**Authors:** Roshni Vamja, Yogesh M, Vijay Vala, Arya Ramachandran, Jay Nagda

**Affiliations:** 1 Department of Community Medicine, M P Shah Medical College, New PG Hostel, MP Shah Medical College Campus, GG Hospital, Patel Colony Post, Jamnagar, Gujarat 361008 India; 2 Department of General Medicine, Shantabaa Medical College and General Hospital, Amreli, India

**Keywords:** MAFLD, Fatty liver, Fatty liver index, Diagnostic accuracy, Metabolic syndrome

## Abstract

**Background:**

Metabolic-associated fatty liver disease (MAFLD) is a major public health problem worldwide. This study aimed to determine the prevalence of MAFLD and evaluate the diagnostic accuracy of the Fatty Liver Index (FLI) compared to ultrasonography for detecting fatty liver in adults attending a tertiary care hospital in Gujarat, India.

**Methods:**

This cross-sectional study included 500 adults visiting the outpatient department between January 2023 and December 2023. MAFLD was diagnosed on ultrasound. FLI was calculated using body mass index, waist circumference, triglycerides, and gamma-glutamyl transpeptidase levels. FLI ≥ 60 indicated fatty liver. Logistic regression analysis identified factors associated with fatty liver.

**Results:**

MAFLD prevalence was 32.2% on ultrasound. High FLI (≥ 60) was present in 26.2%. Male sex, higher BMI, waist circumference, night shift work, diabetes, and triglycerides were independent predictors of fatty liver. FLI showed excellent diagnostic accuracy with a sensitivity of 96%, specificity of 92.5%, and AUC of 0.92 for detecting fatty liver on ultrasound.

**Conclusion:**

MAFLD prevalence among adults was high in this hospital-based sample. FLI can serve as an accurate non-invasive tool for identifying individuals with a high probability of MAFLD. These findings emphasize the need for larger population-based studies and the implementation of regular MAFLD screening programs in high-risk groups.

## Introduction

Metabolic-associated fatty liver disease (MAFLD) is emerging as the leading cause of chronic liver disease worldwide. MAFLD is considered the hepatic manifestation of metabolic syndrome and is closely associated with obesity, insulin resistance, hypertension, and dyslipidemia [1]. It encompasses a spectrum of conditions characterized by hepatic steatosis in the absence of secondary causes such as alcohol use, medications, or monogenic disorders [2]. The disease spectrum ranges from simple steatosis to metabolic-dysfunction-associated steatohepatitis (MASH), progressive fibrosis, cirrhosis, and hepatocellular carcinoma [3]. The global overall prevalence of MAFLD was 38.77% (95% CI 32.94% to 44.95%) [4]. Whereas the pooled overall prevalence of NAFLD in the general population in the South Asian population was 26.9% (95% CI: 18.9–35.8%) [5]. In India, the estimated pooled prevalence was 38.6% (95% CI 32–45.5) among the adult population [6].

Current management guidelines for NAFLD/MAFLD at the primary care level in India recommend lifestyle modifications, including weight loss and increased physical activity, as first-line interventions. Pharmacological therapy is considered for patients with more advanced diseases or those who fail lifestyle interventions [7].

Liver biopsy is the gold standard test for diagnosis and staging of MAFLD. However, its invasive nature, cost, sampling errors, and procedure-related complications restrict its use for mass screening [8]. Imaging modalities like ultrasound, CT, and MRI can detect fatty infiltration but cannot differentiate simple steatosis from MASH or assess fibrosis. Vibration-controlled transient elastography like Fibroscan can estimate liver stiffness and fibrosis but has limited accuracy for lesser degrees of fibrosis [9]. Serum biomarkers and scores based on clinical and laboratory parameters have been developed as non-invasive tools for evaluating MAFLD.

Fatty Liver Index (FLI) is a simple algorithm derived from body mass index, waist circumference, triglycerides, and gamma-glutamyl transpeptidase (GGT) levels. It has been validated to detect fatty liver in various populations with good accuracy [10]. While FLI has been previously used to predict NAFLD, its application to MAFLD is novel. MAFLD has broader diagnostic criteria that include metabolic dysfunction, which may affect the predictive accuracy of FLI. With this context, the present study aimed to determine the prevalence of MAFLD and evaluate the diagnostic performance of the Fatty Liver Index (FLI) compared to ultrasonography for detecting fatty liver in adults attending a tertiary care hospital in Gujarat, India. With the rising burden of obesity and metabolic syndrome, understanding the epidemiology of MAFLD and identifying accurate non-invasive screening tools is crucial for early detection and intervention strategies.

## Materials and methods

### Study design and setting

This is a prospective cross-sectional diagnostic accuracy study conducted between January 2023 and December 2023 at Tertiary Care Hospital in Gujarat, India.

### Study population

The study population included 500 adults The study population included adults aged 18 years and above visiting the general medicine outpatient department. Participants were selected based on the presence of at least one metabolic risk factor (obesity, diabetes, hypertension, or dyslipidemia) identified during routine clinical assessment.

### Sample size calculation

For the diagnostic accuracy component, we used the formula for paired proportions (McNemar’s test), Taking a sensitivity of 61%, and specificity of 86%, with a desired precision of ± 5% and alpha of 0.05, which yielded a required sample size of 366. We increased this to 500 to account for potential dropouts and to improve precision [11].

### Sampling-technique

**Fig. 1:**
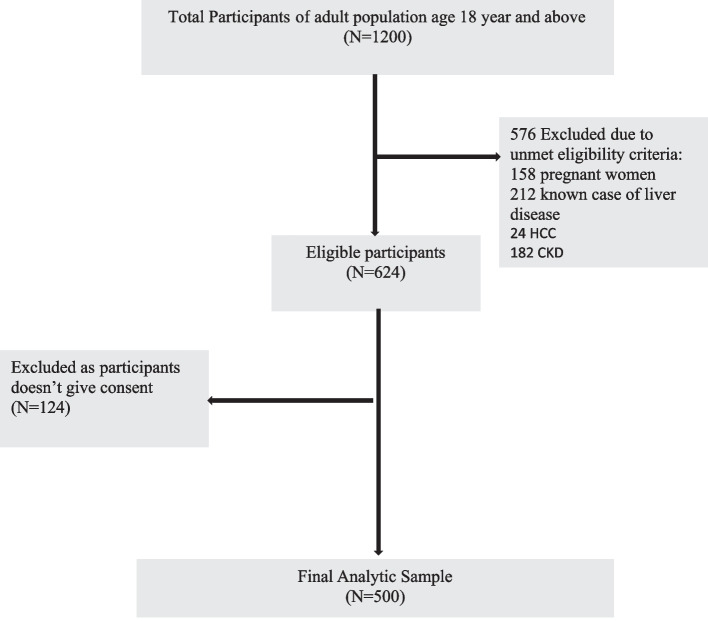
Flowchart for participant recruitment process

Consecutive sampling was used. All eligible adults visiting the general medicine outpatient department during the study period were screened and recruited until the target sample size was achieved (Fig. 1).

### Inclusion criteria

- Age ≥ 18 years
- Provided informed consent

### Exclusion criteria

- Pregnant women
- Known liver disease other than MAFLD (viral hepatitis, autoimmune liver disease, etc.)
- Decompensated cirrhosis
- Hepatocellular carcinoma
- Heart failure
- Chronic kidney disease stage 3 or higher

### Data collection tool

A predesigned questionnaire collected information on demographic details, lifestyle factors, clinical history, and anthropometric measurements. Fasting blood samples were collected for lipid profile. Transient elastography (Fibro Scan) was performed to assess the degree of fibrosis. Ultrasonography of the abdomen was done to diagnose fatty liver. A probe transducer emits vibrations that propagate through the liver tissue. The velocity of the wave is measured and expressed as liver stiffness measurement in kilopascals (kPa). Higher scores indicate increasing fibrosis. Scores < 7.0 kPa are normal, 7.1–9.4 kPa indicate moderate fibrosis and ≥ 9.5 kPa advanced fibrosis [12].

Ten valid measurements were obtained for each patient. All measurements were taken in a single sitting by trained personnel using standardized procedures. The median value was taken as representative of liver stiffness after excluding invalid measurements. Liver stiffness measurement was considered reliable only if the interquartile range to median value ratio was ≤ 30% and the success rate was ≥ 60% [13].

Metabolic syndrome was defined as per the Harmonizing criteria which includes any three of the following—increased waist circumference, elevated triglycerides ≥ 150 mg/dl, reduced HDL < 40 mg/dL, cholesterol, hypertension (SBP > 140, DBP > 90) and elevated fasting glucose ≥ 126 mg/dl [14, 15]. Data on medication history and the total number of medications was collected from medical records and prescriptions. Gallstone disease was diagnosed by experienced radiologists using ultrasonography (Acuson, Sequoia 512, Siemens, Mountain View) after the subjects had fasted for at least 8 h. Gallstone disease was defined as the ultrasonographic presence of gallstones or absence of the gallbladder on ultrasonography due to a previous history of cholecystectomy [16]. Gallstones were diagnosed based on the presence of movable hyper-echoic foci with acoustic shadows. This definition was included to assess the association between gallstone disease and fatty liver, as suggested by recent literature [16, 17].

### Assessment of fatty liver

Fatty liver was diagnosed on ultrasound based on standard criteria—increased echogenicity of the liver compared to the kidney, vascular blurring, and deep attenuation of the ultrasound signal. MAFLD was diagnosed on ultrasound based on the presence of hepatic steatosis in addition to one of the following criteria: overweight/obesity, presence of type 2 diabetes mellitus, or evidence of metabolic dysregulation [18]. Ultrasound examinations were performed by experienced radiologists (> 5 years experience) using a high-resolution B-mode ultrasonography system. MAFLD was diagnosed based on the presence of increased echogenicity of the liver compared to the kidney, vascular blurring, and deep attenuation of the ultrasound signal. To ensure reliability, 10% of scans were independently reviewed by a second radiologist, with an inter-observer agreement of κ = 0.85.

Fatty Liver Index (FLI) was calculated using the published formula incorporating BMI, waist circumference, triglycerides, and GGT levels. FLI ≥ 60 was defined as fatty liver [19].

$$\frac{\mathrm{FLI}\:=\:\mathrm e0.953\;\_\;\log\;\mathrm e\;(\mathrm{triglycerides})\;0.139\;\_\;\mathrm{BMI}\;0.718\;\_\;\log\;\mathrm e\;(\mathrm{GGT})\;0.053\_\mathrm{waist}\;\mathrm{circumference}\;\_\;15.745}{1\:+\:\mathrm e0.\;953\;\_\;\log\;\mathrm e\;(\mathrm{triglycerides})\;139.0\;\_\;\mathrm{BMI}\;0.718\;\_\;\log\;\mathrm e\;(\mathrm{GGT})\;0.053\;\_\;\mathrm{waist}\;\mathrm{circumference}\;15.745\}\;\ast\;100}$$

### Statistical analysis

All data was analyzed using SPSS version 20.0 (IBM Corp, Armonk, NY). Continuous variables were expressed as mean ± standard deviation or median (interquartile range) based on the distribution. Categorical variables were expressed as frequency and percentage.

The prevalence of MAFLD diagnosed by ultrasound was calculated. The Fatty Liver Index (FLI) was calculated for each participant based on the published formula using BMI, waist circumference, triglycerides, and GGT levels. Participants were categorized as having low (< 30), intermediate (30–59), and high (≥ 60) FLI.

Logistic regression was used to examine factors associated with high (≥ 60) versus low (< 30) fatty liver index. Age was categorized in 5-year increments. Variables with *p* < 0.05 in univariate analysis were included in the multivariate model. Adjusted odds ratios with 95% confidence intervals were calculated.

Sensitivity, specificity, positive predictive value (PPV), and negative predictive value (NPV) were calculated to assess the diagnostic performance of FLI compared to ultrasound. True positives (TP), false positives (FP), true negatives (TN), and false negatives (FN) were determined based on FLI ≥ 60 cutoff and ultrasound diagnosis. Receiver operating characteristic (ROC) curve analysis was performed to identify the optimal cutoff value of FLI by plotting sensitivity versus 1-specificity. The area under the ROC curve (AUC) was calculated to assess the predictive accuracy of FLI.

All tests were two-tailed and a *p*-value < 0.05 was considered statistically significant.

### Ethical considerations

The study protocol was approved by the Institutional Ethics Committee of Tertiary Care Hospital (REF No:258/03/2023). Written informed consent was obtained from all participants before enrolment. Patient confidentiality was maintained using unique identification codes.

## Results

Table 1 describes the characteristics of the 500 participants in the study. The mean age was 45.2 years, with 242 males (48.4%) and 258 females (51.6%). The mean BMI was 28.5 kg/m2, and the mean waist circumference was 92.4 cm. 212 (42.4%) worked night shifts. The mean sleep duration was 6.8 h. 152 (30.4%) were current or past smokers. The prevalence of diabetes was 142 (28.4%), hypertension 154 (30.8%), and ischemic heart disease 51 (10.2%). Mean triglyceride level was 1.8 mmol/L, HDL 1.2 mmol/L, and LDL 3.1 mmol/L. Metabolic syndrome was present in 47 (9.4%) participants. Gallstone disease and prior cholecystectomy were reported in 67 (13.4%) and 56 (11.2%) respectively.

**Table 1:** Characteristics of the study population (*n* = 500)

Variable	n (%) or mean ± SD
Age (years)	45.2 ± 12.3
Sex	
Male	242 (48.4%)
Female	258 (51.6%)
BMI (kg/m2)	28.5 ± 4.1
Waist circumference (cm)	92.4 ± 10.5
Daily working hours	8.5 ± 1.2
Working schedule	
Day shift	288 (57.6%)
Night shift	212 (42.4%)
Sleep duration (hours)	6.8 ± 1.1
Current/past smoker	152 (30.4%)
Diabetes	142 (28.4%)
Hypertension	154 (30.8%)
IHD	51 (10.2%)
Stroke	24 (4.8%)
HTN + DM	38 (7.6%)
HTN + IHD	26 (5.2%)
HTN + DM + Stroke	13 (2.6%)
Triglycerides (mmol/L)	1.8 ± 1.1
HDL (mmol/L)	1.2 ± 0.3
LDL (mmol/L)	3.1 ± 0.9
Metabolic syndrome	47 (9.4%)
History of Gallstone	67 (13.4%)
History of cholecystectomy	56 (11.2%)

*BMI* Body Mass Index, *IHD* Ischemic Heart Disease, *HTN* Hypertension, *DM* Diabetes Mellitus, *HDL* High-Density Lipoprotein, *LDL* Low-Density Lipoprotein

Table 2 shows the prevalence of fatty liver based on Fatty Liver Index (FLI) categories. 40% had low FLI, 34% intermediate, and 26% high. It also shows the prevalence of MAFLD on ultrasound was 32.2%, while high FLI was present in 26.2%. The most common causes of high FLI were overweight/obesity in 18.4%, alcohol use in 16.6%, diabetes in 7.2%, and dyslipidemia in 11.6%.

**Table 2:** Prevalence of fatty liver by fatty liver index category and ultrasound

Fatty liver index	n (%)
Low (< 30)	197 (39.4%)
Intermediate (30–59)	172(34.4%)
High (≥ 60)	131(26.2%)
MAFLD by ultrasound	161(32.2%)

Table 3 displays the factors associated with high versus low fatty liver index in univariate and multivariate logistic regression. Male sex (adjusted OR 1.74, 95% CI 1.13–2.68, *p* < 0.05), higher BMI (AOR 1.80 per 1 kg/m2, 95% CI 1.04–2.96, *p* < 0.05), larger waist circumference (AOR 2.48 per 10 cm, 95% CI 1.28–5.71, *p* < 0.05), night shift work (AOR 2.52, 95% CI 1.98–8.35, *p* < 0.01), shorter sleep duration (AOR 0.91 per hour, 95% CI 0.82–0.98, *p* < 0.01), smoking (AOR 1.62, 95% CI 1.06–2.47, *p* < 0.05), diabetes (AOR 3.48, 95% CI 1.79–8.77, *p* < 0.01), hypertension (AOR 2.42, 95% CI 1.89–6.27, *p* < 0.05), stroke (AOR 2.06, 95% CI 1.92–4.62, *p* < 0.05), hypertension with diabetes (AOR 3.79, 95% CI 1.96–8.35, *p* < 0.01), hypertension with IHD (AOR 2.06, 95% CI 1.97–4.38, *p* < 0.05), higher triglycerides (AOR 1.28 per mmol/L, 95% CI 1.12–1.46, *p* < 0.05), lower HDL (AOR 0.68 per mmol/L, 95% CI 0.47–0.98, *p* < 0.01), higher LDL (AOR 1.42 per mmol/L, 95% CI 1.16–1.73, *p* < 0.05), metabolic syndrome (AOR 3.62, 95% CI 1.04–5.05, *p* < 0.01), gallstone disease (AOR 1.98, 95% CI 1.81–3.25, *p* < 0.05), and prior cholecystectomy (AOR 1.86, 95% CI 1.87–3.97, *p* < 0.05) were associated with high fatty liver index.

**Table 3:** Factors associated with fatty liver index

Variable	High vs. Low fatty liver index	
	**Crude OR (95% CI)**	**Adjusted OR (95% CI)**
Age (per 5 years)	1.21 (1.05–1.39)	1.14 (0.98–1.33)
Male sex	1.86 (1.21–2.87)	1.74 (1.13–2.68)*
BMI (per 1 kg/m2)	1.12 (1.07–1.18)	1.80 (1.04–2.96)*
Waist circumference (per 10 cm)	2.52 (1.32–4.75)	2.48 (1.28–5.71)*
Night shift work	2.61 (1.05–4.47)	2.52 (1.98–8.35)**
Sleep duration (per 1 h)	0.88 (0.79–0.97)	0.91 (0.82–0.98)**
Current/past smoker	1.69 (1.12–2.55)	1.62 (1.06–2.47)*
Current/past alcohol use	4.86 (1.21–8.85)	3.74 (1.13–6.69)**
Diabetes	3.79 (1.98–6.26)	3.48 (1.79–8.77)**
Hypertension	2.61 (1.02–2.54)	2.42 (1.89–6.27)*
IHD	1.86 (0.92–3.74)	1.62 (0.79–3.35)
Stroke	2.47 (1.12–5.44)	2.06 (1.92–4.62)*
HTN + DM	2.12 (1.15–3.91)	3.79 (1.96–8.35)**
HTN + IHD	2.35 (1.12–4.91)	2.06 (1.97–4.38)*
Triglycerides (per 1 mmol/L)	1.35 (1.19–1.53)	1.28 (1.12–1.46)*
HDL (per 1 mmol/L)	0.61 (0.43–0.88)	0.68 (0.47–0.98)**
LDL (per 1 mmol/L)	1.52 (1.25–1.85)	1.42 (1.16–1.73)*
Metabolic syndrome	2.79 (1.15–5.77)	3.62 (1.04–5.05)**
History of Gallstone	1.74 (0.88–3.45)	1.98 (1.81–3.25)*
History of Cholecystectomy	1.95 (0.92–4.13)	1.86 (1.87–3.97)*

Multivariate logistic regression analysis adjusted for age, gender, BMI, waist circumference, work schedule, sleep duration, smoking, alcohol use, diabetes, hypertension, ischemic heart disease, stroke, metabolic syndrome, and dyslipidemia

*OR* Odds Ratio, *CI* Confidence Interval, *BMI* Body Mass Index, *IHD* Ischemic Heart Disease, *HTN* Hypertension, *DM* Diabetes Mellitus, *HDL* High-Density Lipoprotein, *LDL* Low-Density Lipoprotein

^*^P-value < 0.05-Significant. **P-value < 0.001-Highly Significant

Table 4 shows FLI had excellent diagnostic accuracy for detecting fatty liver on ultrasound with sensitivity 96% (95% CI 91.1–98.4%), specificity 92.5% (95% CI 86.7–96.2%), PPV 92.4% (95% CI 86.5–96.2%), NPV 96.1% (95% CI 90.8–98.7%), and accuracy 94.6% (95% CI 91.9–96.6%).

**Table 4:** Performance of fatty liver index for predicting ultrasound-diagnosed fatty liver

Measure	Estimate (95% CI)
Sensitivity	96.0% (95% CI: 91.1%-98.4%)
Specificity	92.5% (95% CI: 86.7%-96.2%)
PPV	92.4% (95% CI: 86.5%-96.2%)
NPV	96.1% (95% CI: 90.8%-98.7%)
Accuracy	94.6% (95% CI: 91.9%-96.6%)

Table 5 shows

- At a cutoff of > 30 FLI, sensitivity of 80% and specificity of 60%
- At > 45 FLI, sensitivity of 90% and specificity of 70%
- At ≥ 60 FLI, the primary cutoff used in our analysis, we had a sensitivity of 96% and specificity of 92.5% based on our observed data.

**Table 5:** FLI thresholds of 30, 45, and 60

Cutoff	1—Specificity	Sensitivity
> 30	0.4	0.8
> 45	0.3	0.9
≥ 60	0.075	0.96

**Fig. 2:**
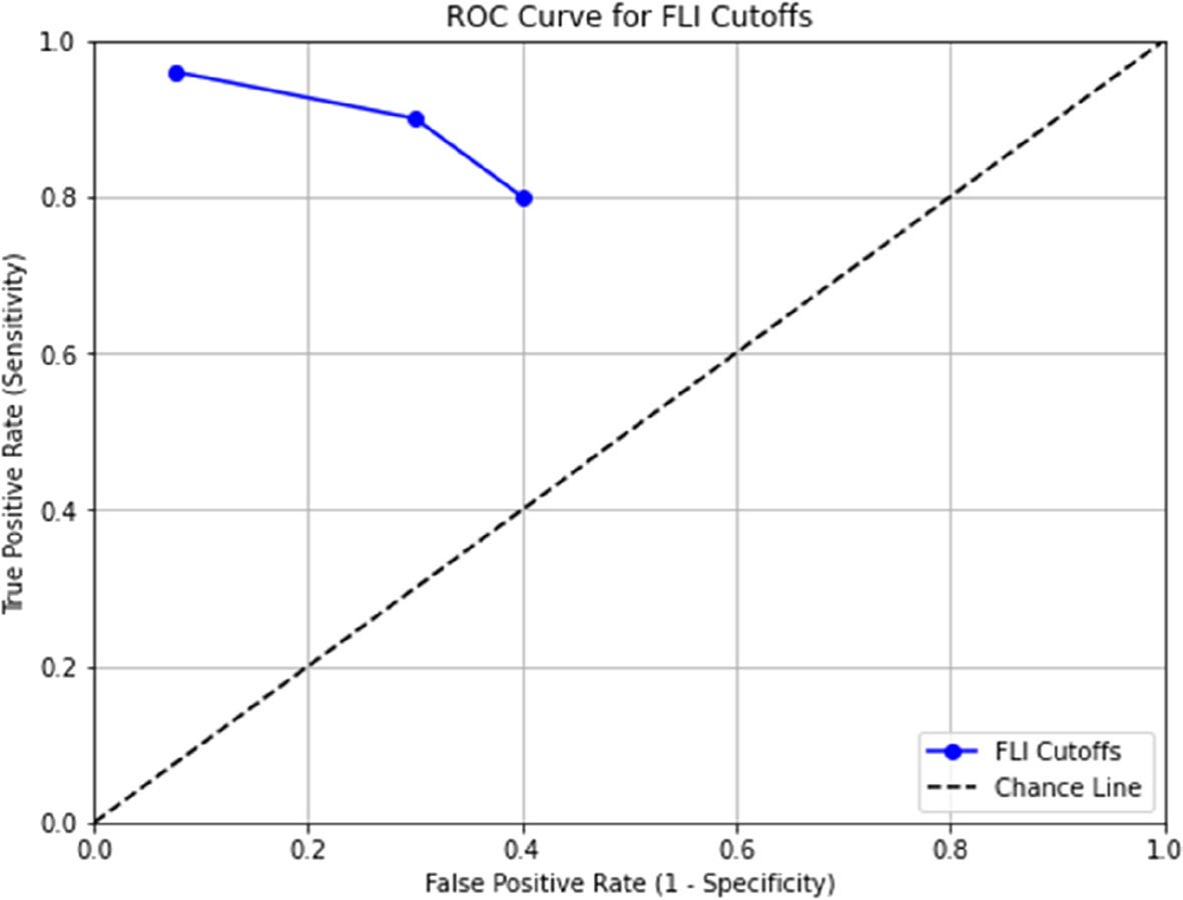
ROC curve for various FLI cutoffs

We generated the ROC curve by plotting sensitivity vs 1-specificity at these different cutoffs. The area under this curve was 0.94, indicating excellent accuracy of FLI ≥ 60 for predicting fatty liver compared to ultrasound (Fig. 2).

In summary, the predefined cutoffs allowed us to categorize fatty liver risk and the multiple thresholds helped plot the ROC curve to assess diagnostic performance. The cutoff of ≥ 60 FLI had the best accuracy versus ultrasound in our study population.

## Discussion

The Present cross-sectional study of 500 adults attending a tertiary care hospital in Gujarat, India, found a prevalence of metabolic-associated fatty liver disease (MAFLD) of 32.2% using ultrasonography. The fatty liver index (FLI) demonstrated excellent diagnostic accuracy for detecting MAFLD, with a sensitivity of 96% and specificity of 92.5% at a cutoff of ≥ 60. This is also comparable to a systemic review that reported a prevalence of MAFLD in Asia (30.5%; 95% CI, 29.0%–31.9%) based on ultrasound findings [20]. Population-based studies from India have found a relatively lower prevalence of 16–32% [21, 22]. The prevalence in our study is higher than that reported in the general population likely due to selection bias as our hospital-based sample had more individuals with components of metabolic syndrome.

The proportion of participants with high FLI ≥ 60 in our study was 26.2%. This is slightly lower than the MAFLD prevalence by ultrasound. This is higher than the previous study which reported the prevalence of FLI-defined NAFLD (FLI ≥ 60) was 19.1% [23]. The discrepancy between FLI-predicted and ultrasound-diagnosed prevalence of MAFLD could be attributed to the older age group in our study population in whom FLI may have lower accuracy.

In evaluating the causes of high FLI in our study, the most common associated factors were overweight/obesity (18.4%), alcohol use (16.6%), diabetes (7.2%), dyslipidemia (11.6%) and polypharmacy (4.4%). Polypharmacy, defined as the concurrent use of 5 or more drugs, is a known risk factor for drug-induced liver injury and MAFLD progression [24]. This corroborates existing evidence that MAFLD occurs in association with features of metabolic syndrome and lifestyle factors like alcohol use [25, 26].

The diagnostic performance of FLI in our study was excellent with a high AUC of 0.92, a sensitivity of 96%, a specificity of 92.5%, and an accuracy of 94.6% for detecting fatty liver on ultrasound. This is consistent with previous studies that have validated FLI and reported AUC ranging from 0.82 to 0.88, sensitivity of 61–86%, and specificity of 71–92% [27–29]. Our findings suggest that FLI may be particularly useful in the Indian population, possibly due to its incorporation of metabolic parameters that are highly relevant to MAFLD. FLI’s high sensitivity and specificity in our study support its potential as a screening tool in primary care settings, especially in resource-limited environments where advanced imaging may not be readily available.

The factors associated with high fatty liver index in this study are consistent with prior research. Male sex was associated with a higher risk of fatty liver, which has been reported in previous studies [30]. Higher BMI and waist circumference have been well-established risk factors for NAFLD in multiple studies [31–35]. Night shift work disrupting circadian rhythms has emerged as a novel risk factor for NAFLD in recent years [36]. This underscores the importance of considering occupational factors in MAFLD risk assessment. Short sleep duration is also associated with an increased risk of NAFLD, likely due to metabolic disturbances [37]. Smoking is known to increase the risk of NAFLD and advanced fibrosis [38]. Diabetes and hypertension have consistently been associated with NAFLD [39–41]. The association between dyslipidemia (elevated triglycerides and lowered HDL) aligns with the well-established role of lipid abnormalities in the pathogenesis of NAFLD [33–35, 42]. A history of gallstone disease and cholecystectomy was associated with NAFLD, which is thought to be related to increased biliary cholesterol secretion in NAFLD [17, 43, 44]. Additionally, our results support the observations of Rong et al., who highlighted the importance of lifestyle factors and metabolic parameters in MAFLD development [45].

Overall, the risk factors for high fatty liver index identified in this study are consistent with established demographic, anthropometric, lifestyle, and metabolic factors associated with MAFLD in prior epidemiologic studies. The findings further establish the utility of fatty liver index as a screening tool for the prediction of MAFLD in this population.

### Limitations

- The sample size of 500 adults, while sufficient for our primary analysis, may limit the generalizability of our prevalence estimates. Larger, population-based studies are needed to provide more accurate estimates of MAFLD prevalence in the general population
- As a single-center study conducted at a tertiary care hospital, there may be selection bias leading to an overestimation of MAFLD prevalence compared to the general population.
- The sensitivity and specificity of ultrasonography for detecting hepatic steatosis are reported to be 60–94% and 66–97% respectively, depending on the degree of fatty infiltration [46]. While MRI techniques offer superior accuracy, ultrasound remains the most widely used first-line imaging modality for MAFLD diagnosis in clinical practice due to its accessibility and cost-effectiveness. Reliance on ultrasound alone for MAFLD diagnosis, while clinically relevant, may not capture the full spectrum of the disease.
- Liver biopsy, the gold standard, was not performed due to ethical and practical constraints.
- Causal inferences are limited due to the cross-sectional study design.
- Information on alcohol consumption was based on self-report, which can lead to underreporting. So, further studies should consider the use of a validated questionnaire (AUDIT-C)
- Other causes of the fatty liver such as medications, viral hepatitis, and autoimmune liver disease may have confounded the diagnosis of MAFLD.
- The fatty liver index has not been validated extensively in older populations which formed a significant proportion of our study sample.

### Recommendations

- Large-scale multi-center studies across diverse settings are required to determine the true population prevalence of MAFLD in the country.
- Advanced imaging modalities and biomarkers should be evaluated further to diagnose the entire spectrum of MAFLD including fibrosis staging.
- Prospective studies must be conducted to establish temporal associations between suspected risk factors and the development of MAFLD.
- Regular screening for MAFLD should be considered in high-risk groups such as those with obesity, diabetes, and metabolic syndrome given the high prevalence.

## Conclusion

This hospital-based study found a high prevalence of MAFLD among adults and demonstrated good diagnostic accuracy of FLI for predicting fatty liver on ultrasound. However, given the study limitations, larger population-based studies are needed to confirm these findings and determine the true prevalence and burden of MAFLD in the general population. While our results suggest potential utility of FLI as a screening tool, further research is required before recommending widespread implementation of MAFLD screening programs.

## Data Availability

The datasets generated and/or analyzed during the current study are not publicly available to protect the privacy of the study participants but are available from the corresponding author upon reasonable request.

## Abbreviations

GGT: Gamma-glutamyl Transferase
Tg: Triglyceride
HDL: High-density Lipoprotein
LDL: Low-density Lipoprotein
FLI: Fatty Liver Index
MASH: Metabolic-dysfunction-associated steatohepatitis
MAFLD: Metabolic-associated fatty liver disease
ROC Curve: Receiver operating characteristic curve
AUC: Area under curve
MetS: Metabolic syndrome
SBP: Systolic Blood Pressure
DBP: Diastolic Blood Pressure
IHD: Ischemic Heart Disease
DM: Diabetes Mellitus
HTN: Hypertension
